# Ectopic expression of a bacterial mercury transporter MerC in root epidermis for efficient mercury accumulation in shoots of Arabidopsis plants

**DOI:** 10.1038/s41598-019-40671-x

**Published:** 2019-03-13

**Authors:** Shimpei Uraguchi, Yuka Sone, Minami Kamezawa, Michi Tanabe, Momoko Hirakawa, Ryosuke Nakamura, Yasukazu Takanezawa, Masako Kiyono

**Affiliations:** Department of Public Health, School of Pharmacy, Kitasato University, 5-9-1 Shirokane, Minato-ku, Tokyo, 108-8641 Japan

## Abstract

For mercury phytoextraction, we previously demonstrated in *Arabidopsis thaliana* that a constitutive and ubiquitous promoter-driven expression of a bacterial mercury transporter MerC fused with SYP121, a plant SNARE for plasma membrane protein trafficking increases plant mercury accumulation. To advance regulation of ectopic expression of the bacterial transporter in the plant system, the present study examined whether *merC-SYP121* expression driven by a root epidermis specific promoter (pEpi) is sufficient to enhance mercury accumulation in plant tissues. We generated five independent transgenic Arabidopsis plant lines (hereafter pEpi lines) expressing a transgene encoding MerC-SYP121 N-terminally tagged with a fluorescent protein mTRQ2 under the control of pEpi, a root epidermal promoter. Confocal microscopy analysis of the pEpi lines showed that mTRQ2-MerC-SYP121 was preferentially expressed in lateral root cap in the root meristematic zone and epidermal cells in the elongation zone of the roots. Mercury accumulation in shoots of the pEpi lines exposed to inorganic mercury was overall higher than the wild-type and comparable to the over-expressing line. The results suggest that cell-type specific expression of the bacterial transporter MerC in plant roots sufficiently enhances mercury accumulation in shoots, which could be a useful phenotype for improving efficiency of mercury phytoremediation.

## Introduction

Phytoextraction is a type of phytoremediation, an environmentally friendly technique to remove contaminants in environments using plant physiological activities. In particular toxic element contamination of soils is a major target of phytoextraction, based on the plant element transport ability from soil^1,2^. For efficient phytoextraction of toxic metal pollution, selecting suitable plants with remarkable abilities of metal transport and tolerance is essential. Wild plants which accumulate exceedingly high levels of metals in the tissues are called metal-hyperaccumulator plants, and such plants are suggested as major options for phytoremediation of metal contamination^2^. However, attempts of phytoextraction using hyperaccumulator plants have been limited to such as cadmium (Cd), zinc (Zn) or arsenic (As) contamination, because it depends on natures of the metal accumulating ability of the hyperaccumulator plants. Another possible approach to develop and extend phytoextraction is molecular breeding of transgenic plants with desirable phenotypes of metal accumulation and/or tolerance. A key to establish such transgenic plants with intended functions is selection and regulation of a transgene.

Among the elements causing environmental pollution in the world, mercury (Hg) contamination has been focused as a major target of transgenic-based phytoremediation^3–8^. Most of the precedent studies utilize a mercuric ion reductase MerA gene and/or an organomercurial lyase MerB gene isolated from Hg-resistant bacteria for phytoremediation^3,4,9^. This approach confers Hg tolerance to plants, however, it does not directly facilitate Hg uptake and accumulation in plants. To overcome the problem, we have applied influx-type bacterial mercury transporters such as MerC to achieve Hg phytoextraction. MerC is a bacterial mercury transporter encoded by *merC* gene, a member of *mer* operons conferring mercury resistance to bacteria^10–13^. A unique feature of MerC among known bacterial toxic element transporters is its mercury uptake activity, whereas many of known bacterial transporters are efflux transporters as ArsB for arsenite resistance^14,15^. Introduction of a bacterial mercury uptake transporter MerC gene to the plant model *Arabidopsis thaliana* enhanced Hg accumulation in the plants exposed to either inorganic-mercury or methylmercury^16,17^. A significance of our engineering is that MerC transporter was over-expressed as a fusion protein with Arabidopsis soluble *N*-ethylmaleimide-sensitive factor protein attachment protein receptor (SNARE) proteins to manipulate subcellular localization of MerC in plant cells. A plant syntaxin (Q-SNARE, a group of SNAREs, with a C-terminal transmembrane domain) SYP121 is a plasma-membrane resident SNARE and functions in vesicle trafficking between the Golgi complex and the plasma membrane^18–20^. MerC fused with SYP121 was substantially localized to the plasma membrane in the Arabidopsis cells, whereas MerC without SYP121 was mainly detected in ER-Golgi fractions^16,21^. The enhanced Hg accumulation in the MerC-SYP121 over-expressing plants^16,17^ is attributed to the SYP121-mediated plasma membrane localized MerC, the bacterial Hg uptake transporter. The MerC-SYP121 over-expressing plants also showed increased Cd accumulation^21^, suggesting potential of the bacterial transporter and SNARE fusion proteins for developing phytoextraction of various toxic elements. Another example of the MerC-SNARE based engineering is Arabidopsis expressing MerC fused with AtVAM3, a different Q-SNARE which is localized to tonoplast^22,23^. MerC-AtVAM3 is localized to tonoplast and is likely to function in vacuolar sequestration of Hg in cytosol, leading to enhance Hg tolerance of the plants^16,21^.

**Figure 1:**
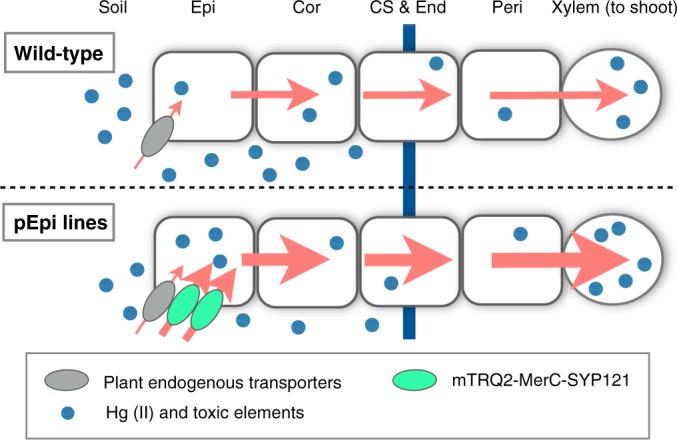
Schematic of root cross sections and radial toxic element transport in the wild-type and pEpi transgenic plants. A hypothesis of this engineering is that epidermis specific expression of mTRQ2-MerC-SYP121 attributed to the epidermis specific promoter pEpi would enhance toxic element like inorganic mercury uptake mediated by plasma membrane localized the MerC fusion proteins. The thicker arrows in the pEpi plants indicate enhanced transport towards inner root vasculatures. Epi, epidermis; Cor, cortex; CS & End, Casparian stripe and endodermis; Peri, pericycle.

These successes of improving Hg uptake and tolerance traits by applying MerC and respective plant SNAREs open up a further possibility of conferring both traits to the plants. For this challenge, cell-type specific regulation of MerC-SNAREs expression is suitable rather than the conventional ubiquitous overexpression approach, because, for instance, ubiquitous expression of MerC-AtVAM3 would increase vacuolar sequestration of toxic metals in root cells, which is likely to decrease radial transport of the target elements toward root vasculatures and eventually reduce their accumulation in shoot. Thus to further advance phytoextraction using the MerC-SNARE fusion proteins, cell-type specific regulation is a major next question that needs to be tackled. The present study reports generation and characterization of Arabidopsis plants expressing MerC-SYP121 specifically in the root surface cells using an Arabidopsis root epidermal cell specific promoter (Fig. 1). Plant roots consist of various well organized types of the cells with different functions. Epidermal cells of the Arabidopsis roots are the outermost cell type contacting soils, thus mediating the first step of elemental uptake by roots. It has been demonstrated for a number of plant nutrient transporters that their expression in the epidermal cells as well as in the endodermis is crucial for proper and efficient nutrient uptake^24–29^. Given the significant roles of the plant nutrient transporters expressed in the epidermis, in this study, we generated Arabidopsis plants expressing MerC-SYP121 fusion proteins in root epidermis and examined a hypothesis whether epidermal specific expression of MerC-SYP121 would be sufficient to improve Hg accumulation ability of the plants.

## Materials and Methods

### Plant materials and growth conditions

*Arabidopsis thaliana* Col-0 ecotype was used as a wild-type control. Col-0 was also used for transformation to obtain *pEpi::mTRQ2-merC-SYP121* transgenic lines (hereafter “pEpi lines”) as described below. The over-expressing line *p35S::merC*-*SYP121* (previously designated as “CS17”, hereafter “p35S line” in this study)^21^ was also used to examine Hg accumulation.

For RNA extraction, agar plates containing one-tenth modified Hoagland medium were used for plant cultivation [100 μM (NH_4_)_2_HPO_4_, 200 μM MgSO_4_, 280 μM Ca(NO_3_)_2_, 600 μM KNO_3_, 5 μM Fe-HBED, 1% sucrose, 5 mM MES, 1.5% (w/v) purified agar (Nacalai Tesque, Kyoto, Japan), pH 5.7]^30,31^. For Hg sensitivity assay, the Hoagland plates containing 25 µM HgCl_2_ were used. For Hg accumulation assay, the following microelements were additionally supplemented to the Hoagland medium (4.63 µM H_3_BO_3_, 32 nM CuSO_4_, 915 nM MnCl_2_, 77 nM ZnSO_4_, 11 nM MoO_3_) and 1.5% purified agar (Nacalai Tesque) or 1% Type A agar (Sigma-Aldrich, St. Louis, MO, USA) was used for short-term and long-term Hg exposure, respectively. For DNA extraction and confocal microscopic analyses, solid plates containing full-strength MS media and 2% sucrose were used for plant cultivation. Arabidopsis seeds (normally 30 to 36 seeds per plate) were surface sterilized and sown on plates containing respective media. After 2 d stratification at 4 °C, plants were grown vertically in a growth chamber LH-411S (Nippon Medical & Chemical Instruments, Osaka, Japan, 16 h light/8 h dark, 100 µmol photons m^−2^ s^−1^ supplied by three-wavelength day white Hf inverter fluorescent lamps, 22 °C).

### Plasmid construction and plant transformation

The primers used for plasmid construction were listed in Supplementary Table S1. A 2,215-bp fragment upstream of the start codon of At5g43030 was amplified from a plasmid pGWB510 harboring the corresponding fragment which was kindly provided by Takehiro Kamiya (University of Tokyo). The upstream sequence of At5g43030 (hereafter pEpi) showed the promoter activity specific to root epidermis and lateral root cap^32^. The obtained pEpi fragment was ligated into the SbfI and XbaI sites of pGWB501^33^. The coding sequence of *mTurquoise2* (*mTRQ2*) was amplified from a plasmid mTurquoise2-C1, a gift from Michael Davidson (Addgene plasmid # 54842). The *merC-SYP121* fragment was amplified from pMACS121.1^21^. The *mTRQ2* and *merC-SYP121* fragments which shared a 15 bp linker sequence in the 3′ and 5′ end of the respective fragments were fused by overlap extension PCR and the resultant fragment *mTRQ2-merC-SYP121* was directionally subcloned into pENTR/D-TOPO (Invitrogen, Carlsbad, CA USA). Then *mTRQ2-merC-SYP121* in pENTR/D-TOPO was subcloned into pGWB501 harboring the pEpi fragment using the LR recombination reaction to obtain *pEpi::mTRQ2-merC-SYP121* (Fig. 1B). The resultant plasmid was introduced into *Agrobacterium tumefaciens* GV3101::pMP90, which were then used for transformation of Col-0 plants by the floral dip method^34^. Transformants were selected on MS agar medium containing hygromycin and T3 homozygous lines were used for the experiments.

### Detection of the T-DNA insertion

Genomic DNA was extracted from leaves as described previously^35^. Obtained DNA was used as template to confirm T-DNA integration into the genome by PCR. Primers specific to *mTRQ2* and *merC* in the T-DNA were used to amplify the T-DNA fragment (Supplementary Table S1). *AtEF1a* (AT5G60390) was also examined as an endogenous reference gene.

### Transcription analyses

To examine *mTRQ2-merC-SYP121* expression in the Arabidopsis transformants, plants grown on the Hoagland agar plates for 7 d were subjected to RNA extraction. RNeasy Plant Mini (QIAGEN, Hilden, Germany) was used for total RNA extraction from roots or shoots. DNase I (QIAGEN) treatment was applied during extraction. PrimeScript RT Master Mix (Takara Bio, Shiga, Japan) was used for cDNA synthesis and quantitative RT-PCR was performed with PowerUp SYBR Green Master Mix (Thermo Fisher Scientific, Carlsbad, CA). *AtEF1a* (At5g60390) served as an internal control. The primer sequences used for expression analyses are listed in Supplementary Table S1.

### Confocal laser microscopy

Arabidopsis plants were grown on the solid MS plates for 4–5 d. Roots were excised from the seedlings and transferred to the one-tenth Hoagland solution containing 4 µM FM4–64. After 3–5 min incubation at room temperature, the roots were observed by a laser scanning confocal microscopy LSM710 (Carl Zeiss, Jena, Germany) with the following excitation and detection wavelengths, respectively: 405 nm and 420–489 nm for mTRQ2; 488 nm and >640 nm for FM4-64.

### Hg accumulation analysis

Two different Hg treatments were applied to examine Hg accumulation ability of pEpi plants compared to the wild-type Col-0 and the overexpressing line p35S: 10 µM HgCl_2_ for 1 d after 10 d growth on control plates and 0.2 µM HgCl_2_ for 12 d from the start of cultivation. The former short-term assay was to assess Hg accumulation of the plants under higher Hg concentrations without severe toxicity. And the latter long-term assay was to evaluate the plant Hg accumulation under environmentally relevant Hg concentrations. The Hg concentrations of the assays were assumed as those of heavily and moderately polluted soils, respectively^36–38^.

For short-term Hg accumulation assay, plants (36 seeds per plate) were grown on the control Hoagland plates for 10 d. Uniformly grown seedlings (30 seedlings per plate, 15 seedlings for each line) were then transferred to plates containing 10 µM HgCl_2_ and grown for an additional day. For long-term Hg accumulation assay, plants (30 seeds per plate, 15 seeds for each line) were germinated and grown on the Hoagland plates containing 0.2 µM HgCl_2_ for 12 d. Roots and shoots from each plate (normally 15 seedlings per line) were separately pooled as a single sample.

At harvest, shoot samples were washed twice with MilliQ water. Root samples were subjected to sequential washing procedures^30^: roots were desorbed for 10 min each in ice-cold MilliQ water, 20 mM CaCl_2_ (twice), 10 mM EDTA (pH 5.7) and MilliQ water. Harvested roots and shoots were dried at 50 °C before acid digestion. Dried plant samples were wet-digested and total mercury concentrations in the digested samples were quantified by a cold vapor atomic absorption spectrometer HG-310 (Hiranuma, Tokyo, Japan) according to the method previously described^16^.

### Statistical analysis

Quantitative RT-PCR data were analyzed by one-way ANOVA, followed by Tukey’s HSD (*P* < 0.05) to verify the statistical significance of differences among the lines. Mercury accumulation and root growth data were analyzed by Student’s t-test to verify the statistical significance of differences between the transformants and the wild-type Col-0 (*P* < 0.05). The R software (ver. 3.5.1) and Microsoft Excel for Mac (ver. 16.16.6) were used for statistical analyses.

## Results and Discussion

### *Generation of* pEpi::mTRQ2-merC-SYP121 *transgenic plants*

**Figure 2:**
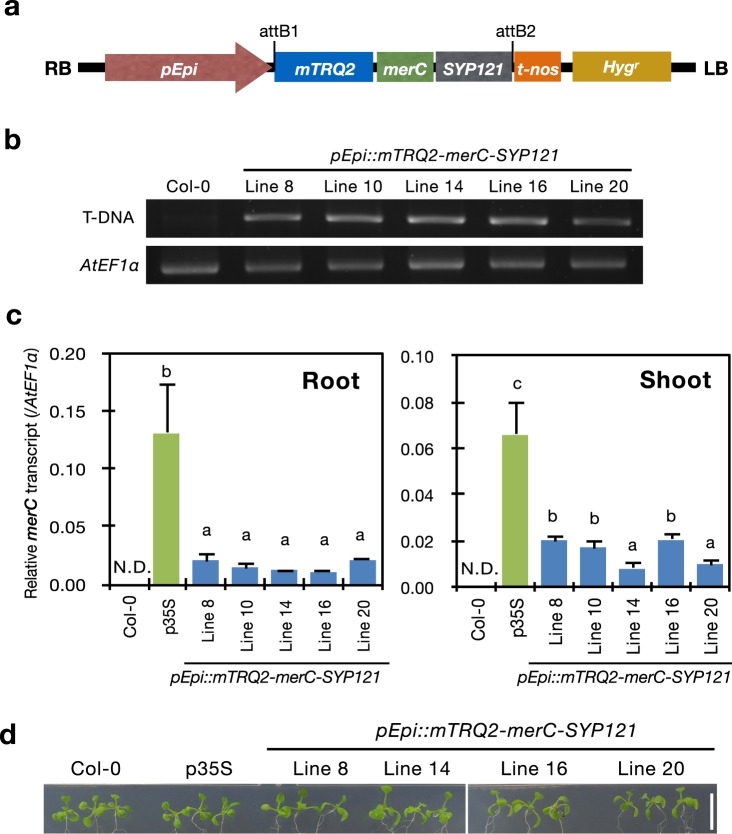
Generation of *pEpi::mTRQ2-merC-SYP121* transgenic Arabidopsis plants. (**a**) Schematic structure of the T-DNA region in the binary vector constructed for generating pEpi transgenic plants. RB, right border; LB, left border; pEpi, putative promoter region of At5g43030; *mTRQ2*, monomeric variant of cyan fluorescent protein (CFP); *merC*, bacterial mercury transporter gene; *SYP121*, plant SNARE gene; t-nos, NOS terminator; Hyg^r^, hygromycin resistance gene. (**b**) Detection of the T-DNA fragment by PCR from genomic DNA of Col-0 (wild-type) and the pEpi transgenic plants. *AtEF1a* served as a reference gene. The full-length gel is presented in Supplementary Fig. S1. (**c**) Quantification of the transgene expression by real-time RT-PCR. Total RNA was extracted from roots and shoots of Col-0 (wild-type), p35S (*merC-SYP121* over-expressing line) and the pEpi lines for cDNA synthesis and subsequent real-time RT-PCR analysis. Expression levels of *merC* were normalized to those of *AtEF1a*. The data are presented as means with standard deviation (*n* = 4). Means sharing the same letter are not significantly different (*P* < 0.05, Tukey’s HSD). (**d**) Phenotypes of Col-0 (wild-type), p35S (*merC-SYP121* over-expressing line) and the pEpi lines grown on the agar plates. A scale bar = 1 cm.

More than 20 independent pEpi lines were obtained in the T1 generation and six of them were suggested as single-copy insertion lines in the T2 generation based on hygromycin resistance, showing segregation ratios close to 3:1 (hygromycin resistance: hygromycin sensitive). Among the six segregating lines, five lines were established as T3 homozygous lines in terms of the T-DNA insertion. Genomic DNA was extracted from leaves of the plants and the T-DNA integration into the genome was examined on these plants by PCR analysis using the specific primers for the T-DNA (Fig. 2a). All the tested pEpi lines (Line 8, 10, 14, 16 and 20) showed a band at the expected size corresponding to the T-DNA fragment although Col-0 did not (Fig. 2b). These results of segregation analysis and T-DNA detection indicated that the obtained transgenic lines had a single copy insertion of the T-DNA in their genome.

Quantitative RT-PCR analysis was then conducted to investigate whether the introduced *mTRQ2-merC-SYP121* gene was expressed in these transgenic lines (Fig. 2c). In addition to the five established pEpi plants, Col-0 and the p35S plants, previously established as the CaMV 35S promoter driven *merC-SYP121* over-expressing line^21^ were examined (Fig. 2c). In the wild-type Col-0 *merC* expression was below the quantification limit of qRT-PCR, whereas the p35S over-expressing line accumulated substantial transcript of *merC* as reported previously^21^. Expression levels of the transgene in the five pEpi lines were approximately 70% or much lower than those of the p35S over-expressing line. In roots, among the pEpi lines, expression levels of the transgene were basically comparable. These results demonstrate that five independent pEpi lines expressing *mTRQ2-merC-SYP121* under the control of the epidermal specific promoter were generated.

In a previous study, *merC-SYP111* transgenic Arabidopsis exhibited abnormal growth defect even under control conditions^21^. Such phenotype is not desirable for application to phytoextraction. However, the pEpi plants grew normally on the agar plates like Col-0 without any visible growth phenotypes (Fig. 2d), indicating that the transgene and its expression does not harm plant growth. We further examined Hg(II) sensitivity of the pEpi plants (Supplementary Fig. S2). Under the Hg(II) condition which reduced Col-0 root growth to 75% of the control, the tested pEpi plants showed similar growth compared to the wild-type Col-0. This suggests that expression of *mTRQ2-merC-SYP121* has little effects on Hg(II) sensitivity of the plants, at least under the tested condition.

### Cell-type specific expression and subcellular localization of mTRQ2-MerC-SYP121

**Figure 3:**
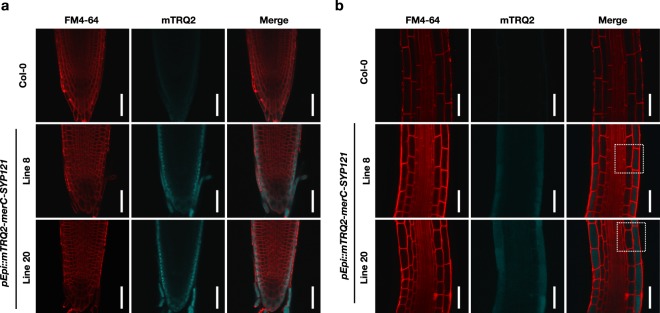
Cell-type specific expression of mTRQ2-MerC-SYP121 in the root tip (**a**) and the elongation zone (**b**) of roots of Col-0 (wild-type) and the pEpi plants (lines 8 and 20). FM4-64 was applied for 3–5 min before laser scanning microscopic analyses. Scale bars = 50 µm. The areas indicated with white dashed lines in the merge images (**b**) are shown with higher magnifications in Fig. 4 for subcellular localization analysis.

**Figure 4:**
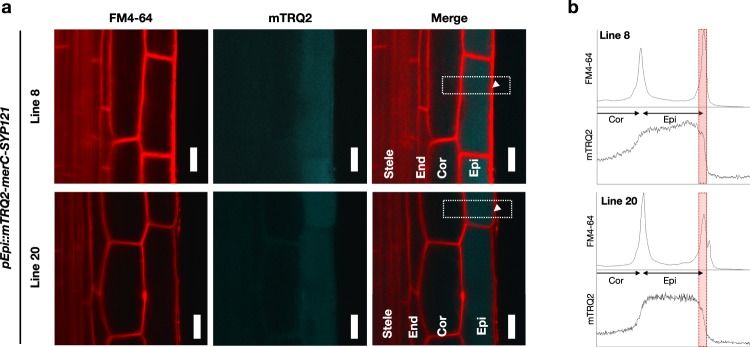
Subcellular localization of mTRQ2-MerC-SYP121 in the roots of the pEpi plants. (**a**) FM4-64 stained plasma membranes and mTRQ2 in the elongation zones of the roots of the pEpi lines 8 and 20. FM4-64 was applied for 3–5 min before laser scanning microscopic analyses. The areas indicated with white dashed lines are subjected to the signal profiling analyses in (**b**). White arrows mark the outer plasma membrane regions (PM_out_) which correspond to the FM4–64 peaks in (**b**) indicated with red dashed lines. Scale bars = 10 µm. Epi, epidermis; Cor, cortex; End, endodermis; Ste, stele. (**b)** Quantification of signal profiles of FM4-64 and mTRQ2 across the epidermal cell regions indicated with white dashed lines in (**a**). Epi, epidermis; Cor, cortex; PMout, outer plasma membrane regions.

Cell-type specific expression of mTRQ2-MerC-SYP121 in the Epi lines was then examined by a confocal laser microscopy (Fig. 3). A fluorescent lipophilic styryl dye FM4-64 was applied as a plasma membrane marker to visualize the cells. Under our microscopic conditions, mTRQ2 signal was not evidently detected in the wild-type Col-0. In the root tips of Line 8 and Line 20, the signal of mTRQ2 was observed in the outermost cell layers, namely lateral root cap in the tip and epidermis in the upper region (Fig. 3a). In the elongation zone which is located above the root tip, mTRQ2 signal was detected specifically in epidermal cells, the outermost cell layer (Fig. 3b). Observation with higher magnification ratios further suggested that the signal of mTRQ2 was specific to epidermis (Fig. 4a). These cell-type specific expression patterns of the pEpi plants were consistent with a previous observation that the promoter activity of pEpi (At5g43030) was specific to lateral root cap and epidermis in roots^32^.

Since the mRNA of *mTRQ2-MerC-SYP121* was also substantially detected in the shoots of pEpi lines (Fig. 2c), we examined mTRQ2 expression in the leaves of pEpi lines (Supplementary Fig. S3). Fluorescence of mTRQ2 was observed in trichomes (leaf hair cells) on the leaf surface (Supplementary Fig. S3a), but not in upper and lower epidermis (Supplementary Fig. S2b,c) and upper and lower mesophyll (Supplementary Fig. S3d,e). This expression pattern in the leaves of the pEpi plants basically coincided with the gene expression patterns of At5g43030 available in the Arabidopsis eFP browser (http://bar.utoronto.ca/efp/cgi-bin/efpWeb.cgi)^39^: trichomes showed some expression of At5g43030 but epidermis and guard cells did not. These results demonstrate that the pEpi transgenic plants express mTRQ2-MerC-SYP121 specifically in the epidermis and lateral root cap in the roots and in the trichomes on the leaves as well.

Subcellular localization of mTRQ2-MerC-SYP121 was also examined (Fig. 4). mTRQ2 signal was detected ubiquitously in the epidermal cells of both Line 8 and Line 20, however, the mTRQ2 signal appeared to colocalize with a plasma membrane marker FM4-64 (Fig. 4a). The colocalization of mTRQ2 and FM4-64 was further examined by image profiling analyses across the endodermal cell regions (Fig. 4b). The mTRQ2 signal was uniformly detected within the epidermal cells of Line 8 and Line 20 but the edges of the mTRQ2 signal were located at the peaks of FM4-64 which indicated the outer plasma membrane. These results suggested that mTRQ2-MerC-SYP121 was at least partially localized to the plasma membrane, whereas majority of mTRQ2-MerC-SYP121 was distributed ubiquitously to other cellular compartments. In our previous studies, transiently expressed GFP-MerC-SYP121 was localized mainly to the plasma membrane in Arabidopsis suspension cultured cells^21^. In a stable expression system, sucrose gradient centrifugation followed by western blot indicated that MerC-SYP121 (without fluorescent proteins) was distributed to the plasma membrane fractions as well as to other fractions like ER-Golgi^16^. These results suggest that fusion with SYP121 functions in MerC targeting to plasma membrane to some degree in plant cells, but the effect would be less in a stably expressing plant system. In the pEpi plants, fusion of mTRQ2, MerC and SYP121, a plasma membrane resident SNARE^18–20^ also contributes to partial plasma membrane localization of MerC fusion proteins. Further analyses of the MerC-SYP121 fusion protein trafficking in plant cells would lead to improve plasma membrane targeting of the MerC fusion protein.

### Mercury accumulation in the pEpi plants

**Figure 5:**
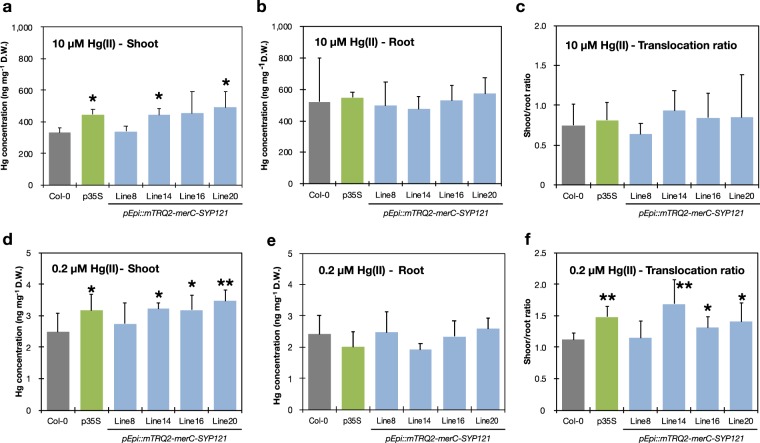
Mercury accumulation in the pEpi plants. (**a**–**c**) Col-0 (wild-type), p35S (*merC-SYP121* over-expressing line) and the pEpi lines were grown on the control agar medium for 10 d and then transferred to the medium containing 10 µM HgCl_2_ for 1 d. Mercury concentrations in shoots (**a**) and roots (**b**) were separately quantified by CV-AAS. Mercury translocation ratios (**c**) were calculated by dividing Hg concentrations of shoots by those of roots. Data represent means with SD (*n* = 4). Asterisks indicate significant differences from the values of Col-0 (*P < 0.05, Student’s two-tailed t test). (**d**–**f**) Col-0, p35S and the pEpi lines were grown on the agar medium containing 0.2 µM HgCl_2_ for 12 d. Mercury concentrations in shoots (**d**) and roots (**e**) and translocation ratios (**f**) were obtained. Data represent means with SD (*n* = 4–6). Asterisks indicate significant differences from the values of Col-0 (*P < 0.05, **P < 0.01, Student’s two-tailed t test).

Finally, Hg accumulation ability of the pEpi plants was examined using the agar-based solid medium system in comparison to the wild-type Col-0 and p35S line (MerC-SYP121 over-expressing plants). The short-term assay was performed to assess relatively early responses of the plants to higher Hg concentrations which can be found in severely polluted sites and the long-term assay was carried out to evaluate the plant Hg accumulation under environmentally relevant moderate Hg concentrations^36–38,40^. First, we examined Hg accumulation in the plants after short-term HgCl_2_ exposure. The p35S plants which over-expressed MerC-SYP121 showed 34% increase of Hg accumulation in shoots in compared to Col-0 (Fig. 5a), confirming the improved Hg accumulation ability under the present condition. Similarly, a number of the pEpi lines also accumulated significantly higher levels of Hg in the shoots compared to the wild-type Col-0 (33% and 48% increase for line 14 and line 20, respectively). No significant difference was detected among the tested lines in root Hg concentrations (Fig. 5b) and translocation ratio from root to shoot (Fig. 5c).

We further examined Hg accumulation ability of the pEpi plants grown under 0.2 µM HgCl_2_ containing medium for 12 d. Even under this lower dose condition, three independent pEpi plants (lines 14, 16 and 20) as well as MerC-SYP121 overexpressing p35S plants exhibited approximately 30% higher Hg concentrations in shoots compared to Col-0 (Fig. 5d). Again expression of MerC fusion proteins did not affect Hg accumulation in roots under the condition (Fig. 5e). Reflecting such Hg distribution patterns, shoot/root Hg ratios were significantly higher in p35S and pEpi transgenic lines (lines 14, 16 and 20) (Fig. 5f).

These results overall suggest that epidermis specific expression of the bacterial Hg transporter MerC fused with SYP121 can confer the same degree of improvement of inorganic Hg accumulation ability to Arabidopsis plants compared to a conventional constitutive and ubiquitous promoter driven expression system^16^. It is noteworthy that mTRQ2-MerC-SYP121 expression in root epidermis enhances Hg accumulation in shoots, represented by higher translocation ratios to shoots of the transgenic plants (Fig. 5f). For nutritional element transporters, it is demonstrated that uptake at epidermal cells is the first and crucial step of root elemental absorption and eventually whole plant nutrient accumulation^24–29^. Our engineering presented in this study also supports the idea that efficient uptake mediated by a transporter expressed at an epidermal cell layer is crucial for plant accumulation of environmental contaminants like mercurials.

In contrast to shoot Hg accumulation, there was no significant difference in root Hg concentrations among the lines (Fig. 5b,e). This could appear unreasonable considering roles of mTRQ2-MerC-SYP121 expression in the root epidermis. However, it is recently suggested that in plant root cells, proper regulation of uptake and efflux transporters with distinct polarities avoids overaccumulation of nutritional elements in root cells^41^. Such efficient element transport towards inner roots would lead to efficient root-to-shoot translocation. Similar accumulation patterns are indeed known for a non-essential element Cd. In high-Cd accumulating rice cultivars, root Cd accumulation does not exceed that of other cultivars but enhanced xylem-mediated root-to-shoot Cd translocation is a major factor for higher shoot Cd accumulation^42^. In several high-Cd rice cultivars, loss-of-function mutation in a vacuolar Cd transporter OsHMA3 gene results in disruption of vacuolar sequestration processes in root cells and eventually increases root-to-shoot translocation and shoot accumulation of Cd^43–45^. Like these cases, although Hg transport mechanisms in plants remain elusive, Hg absorbed at the epidermis by mTRQ2-MerC-SYP121 would be efficiently transported to xylem vessels and subsequently to shoots, which could result in the stable root Hg concentrations among the lines. Nevertheless the enhanced Hg translocation activity from roots to shoots of the pEpi plants would be a useful phenotype for phytoremediation.

### Potential of epidermis specific MerC-SYP121 expression

A significance of the transgenic plants presented here is that mTRQ2-MerC-SYP121 expression in the root epidermis enhances shoot Hg accumulation both under higher/severe and lower/moderate Hg conditions. The Hg concentrations employed in the long-term and short-term assays are assumed as Hg levels often reported for various contaminated sites and those observed in severely polluted soils, respectively^36–38,40^. Our results indicate potentials of the pEpi plants for remediating soils with ranged Hg concentrations, although further experiments are needed under more environmentally relevant conditions.

Moreover, we demonstrate herein that a single-cell layer expression of MerC-SYP121 in roots is effective to enhance plant Hg accumulation. To further facilitate MerC-based phytoextraction of mercurials, multi-cell layer expression of MerC-SYP121 would be a next option. In addition to epidermis, endodermis is another key cell type in roots regulating element transport toward inner root vasculatures because the Casparian strip diffusion barrier exists in the endodermal layer^46,47^. Since effects of MerC-SYP121 expression in the epidermis on enhancing Hg accumulation was comparable to those of ubiquitous over-expression (Fig. 5), expression of MerC-SYP121 both in epidermis and endodermis would be expected to enhance Hg uptake activity of roots more efficiently than the ubiquitous overexpression system. Another possible engineering of such cell-type limited expression of MerC is to introduce an additional transgene for vacuolar sequestration of toxic metals in shoots. Our previous studies show that overexpression of MerC fused with another SNARE, AtVAM3 increased tolerance to mercurials and Cd^16,17,21^. Like SYP121, AtVAM3 is a syntaxin classified into Qa-SNARE but is localized to tonoplast^22,23^. MerC-AtVAM3 fusion proteins were localized to tonoplast^21^ and that subcellular localization is likely to attribute to enhanced Hg tolerance. However, due to its tonoplast localization, ubiquitous overexpression of MerC-AtVAM3 in roots may interfere Hg transport toward inner roots and eventually to shoots, which is a disadvantage for phytoremediation^17^. Shoot specific expression of MerC-AtVAM3 may confer Hg tolerance to plants without affecting uptake in roots whereas root specific expression of MerC-SYP121 facilitates root Hg uptake. Further works are needed to generate such transgenic plants and examine the hypothesis for efficient Hg phytoremediation.

## Supplementary information

Supplementary information
