# Supplementary material for: Ectopic expression of a bacterial mercury transporter MerC in root epidermis for efficient mercury accumulation in shoots of Arabidopsis plants
Source: Sci Rep. 2019 Mar 13;9:4347. doi: 10.1038/s41598-019-40671-x (PMC6416403; doi:10.1038/s41598-019-40671-x)
Supplement: Supplementary file 1 — Supplementary information [file 41598_2019_40671_MOESM1_ESM.pdf]

Supplementary information

Ectopic expression of a bacterial mercury transporter MerC in root epidermis for efficient mercury accumulation in shoots of Arabidopsis plants

Shimpei Uraguchi[1], Yuka Sone[1], Minami Kamezawa[1], Michi Tanabe[1], Momoko Hirakawa[1], Ryosuke Nakamura[1], Yasukazu Takanezawa[1], Masako Kiyono[1]*

[1] Department of Public Health, School of Pharmacy, Kitasato University, 5-9-1 Shirokane, Minato-ku, Tokyo 108-8641, Japan

Table S1 List of primers used in the study

Primer set for		Primer name	Primer sequence (5' to 3')
Plasmid construction	promoter	Sbf1-pEpi_Fw	aattcctgcaggGTTGTGTTGTATATTGTTG
		Xba1-pEpi_Rv	ctagtctagaTAGCTAGCTAATGGTAGA
	mTRQ2	XFPs_Fw	caccATGGTGAGCAAGGGCGAG
		XFPs_Rv	ggctcctcctcctccCTTGTACAGCTCGTCCATGC
	merC-SYP121	merC_Fw	ggaggaggaggagccATGGGACTGATGACACGC
		SYP121_Rv	TCAACGCAATAGACGCCTTG
Genotyping	T-DNA	XFPs_Fw	caccATGGTGAGCAAGGGCGAG
		merC_Rv2	TTTTATCGGCAATGCGTGTC
	AtEF1a	AtEF1a Fw	AGCACGCTCTTCTTGCTTTC
		AtEF1a Rv	AACGCCTGTCAATCTTGGTC
Real-time PCR	*merC*	merC Fw2	CTTCTTGAGCCAGTACGAGG
		merC Rv3	GCCATTGCCGATGACTGAAC
	AtEF1a	AtEF1a Fw2	TGCCACACCTCTCACATTGC
		AtEF1a Rv2	CAACGCCTGTCAATCTTGGTC

Lowercase sequences indicate additional sequences for restriction enzyme digestion (promoter), TOPO-directional cloning (XFPs_FW) or adding linker sequences (XFPs_Rv and merC_Fw).

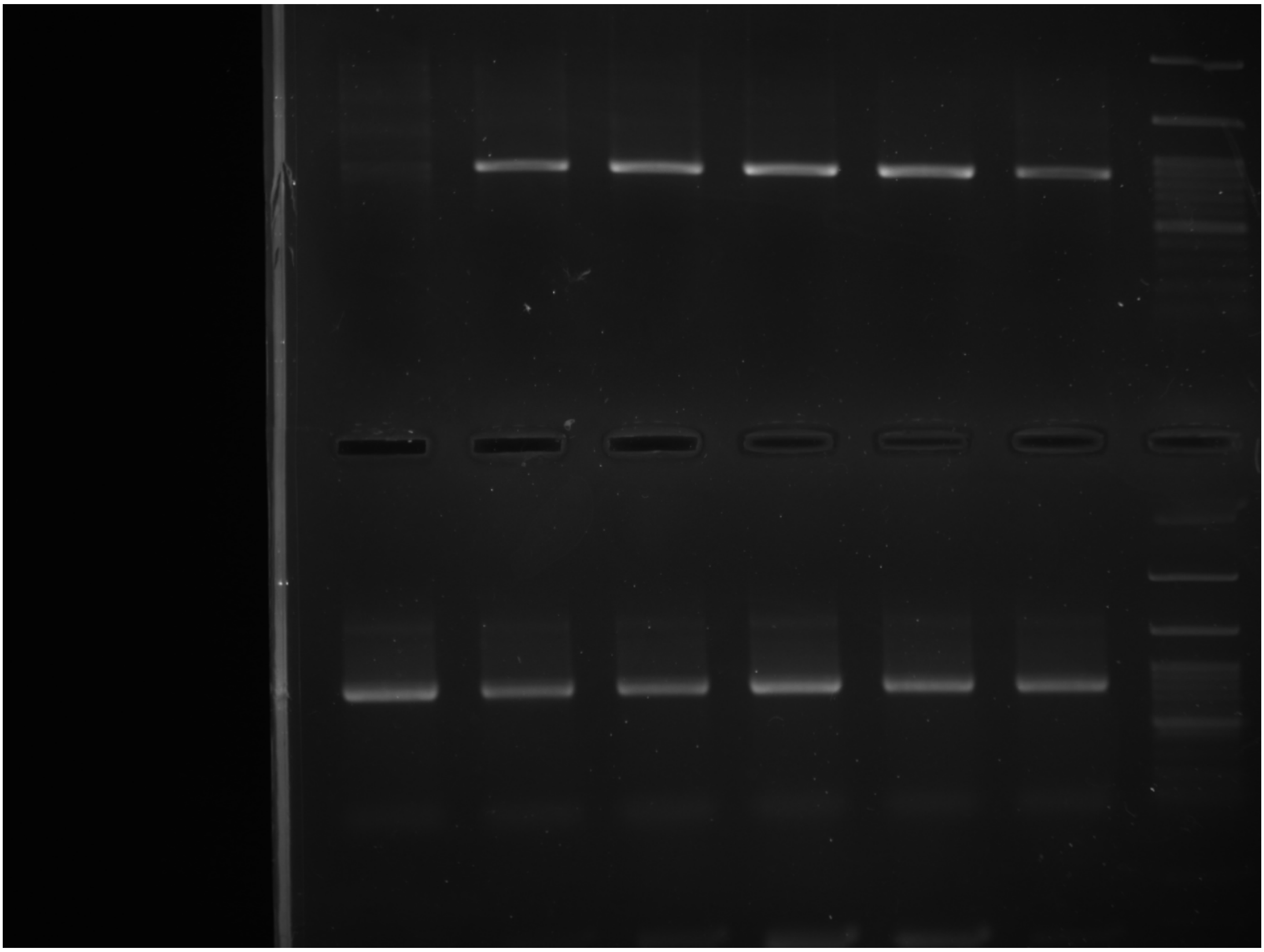

Supplementary Fig. 1 Full-length image of agarose gel used for Figure 2 b.

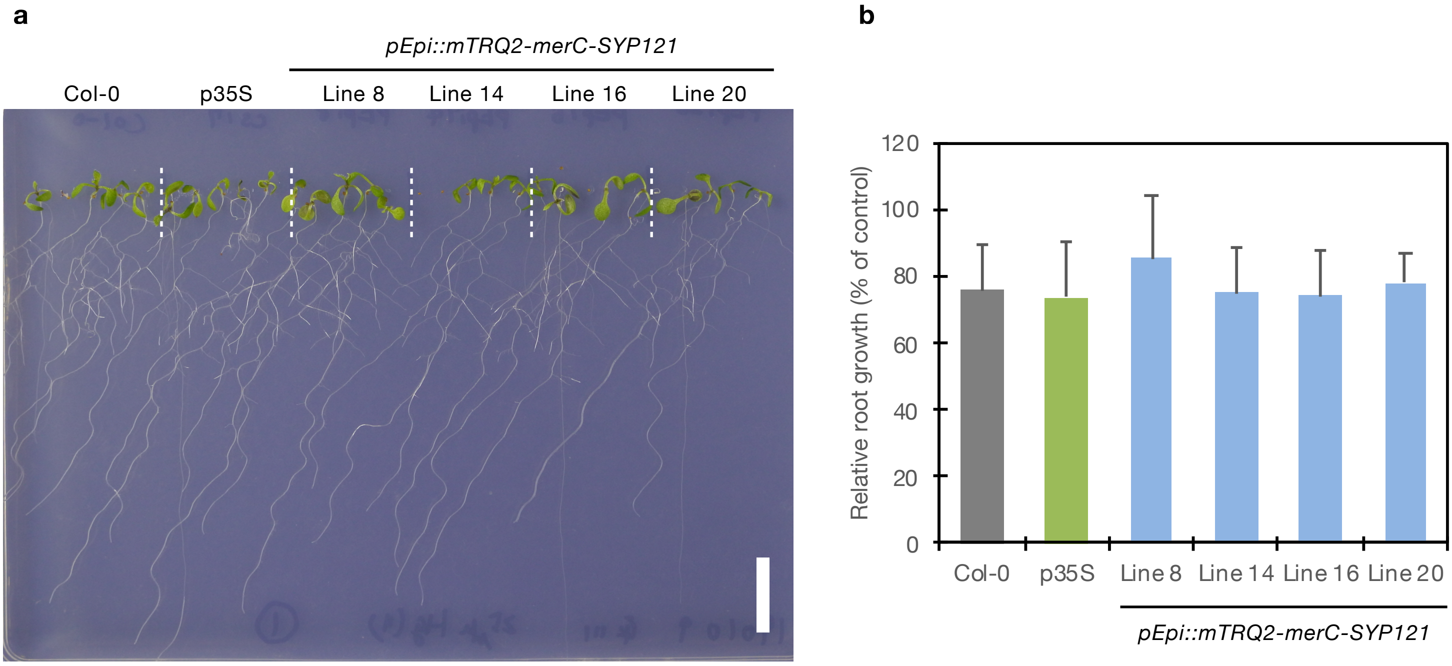

Supplementary Fig. 2 Hg(II) sensitivity assay of the pEpi plants.

a Phenotypes of Col-0, p35S, and the pEpi lines grown on the agar medium containing 25 μM Hg(II) for 12 d. Scale bar = 1 cm. **b** Relative primary root length of the seedlings grown on the agar medium containing 25 μM Hg(II) for 12 d. Values are shown as a percentage of each control. Data represent means with the SD from two independent experiments (*n* = 14-27). There is no significant difference between Col-0 and the transgenic lines according to Student's t test.

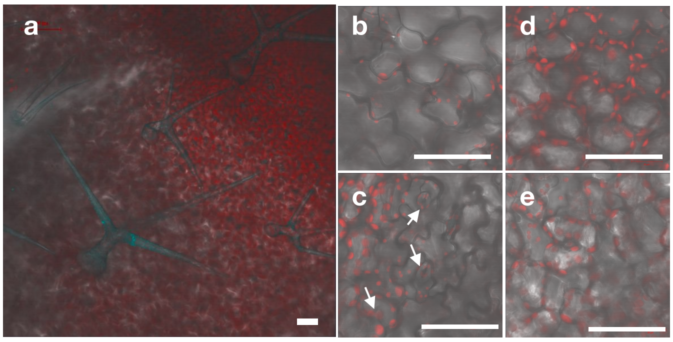

Supplementary Fig. 3 Microscopic analyses of mTRQ2-MerC-SYP121 expression in leaves of the pEpi plants (Line 16). **a** upper epidermis and trichomes. **b** upper epidermis. **c** Lower epidermis and stomata (indicated with white arrows). **d** Upper mesophyll. **e** Lower mesophyll. Red signal indicates autofluorescence of chloroplast. Scale bars = 50 µm.
